# [^18^F]-AV-1451 binding in the substantia nigra as a marker of neuromelanin in Lewy body diseases

**DOI:** 10.1093/braincomms/fcab177

**Published:** 2021-08-28

**Authors:** Elijah Mak, Antonina Kouli, Negin Holland, Nicolas Nicastro, George Savulich, Ajenthan Surendranathan, Maura Malpetti, Roido Manavaki, Young T Hong, Tim D Fryer, Franklin Aigbirhio, James B Rowe, John T O’Brien, Caroline H Williams-Gray

**Affiliations:** 1 Department of Psychiatry, University of Cambridge, Cambridge CB22QQ, UK; 2 Department of Clinical Neurosciences and Cambridge University Hospital NHS Trust, University of Cambridge, Cambridge CB20SZ, UK; 3 Department of Clinical Neurosciences, Geneva University Hospitals, Geneva, Switzerland; 4 Department of Radiology, University of Cambridge, Cambridge CB22QQ, UK; 5 Wolfson Brain Imaging Centre, University of Cambridge, Cambridge CB22QQ, UK; 6 Medical Research Council Cognition and Brain Sciences Unit, University of Cambridge, Cambridge CB37EF, UK

**Keywords:** Lewy bodies, dementia, tau, neuromelanin, Parkinson's disease

## Abstract

While [^18^F]-AV-1451 was developed as a PET radiotracer with high affinity for hyperphosphorylated tau, it has been proposed that loss of ‘off-target’ [^18^F]-AV-1451 binding to neuromelanin in the substantia nigra could be a surrogate marker of Lewy body diseases. [^18^F]-AV-1451 binding was measured in the substantia nigra of patients with Parkinson’s disease (*n* = 35), dementia with Lewy bodies (*n* = 10) and separate control groups (*n* = 37; *n* = 14). Associations with motor symptoms, cognition and disease duration were evaluated using linear regression models. The dementia with Lewy bodies group had significantly reduced substantia nigra [^18^F]-AV-1451 binding compared to controls after adjusting for age (*P* < 0.05). However, there were no significant differences in substantia nigra [^18^F]-AV-1451 binding between Parkinson’s disease and controls. Substantia nigra [^18^F]-AV-1451 binding was not associated with age, disease duration, Movement Disorders Society—Unified Parkinson’s Disease Rating Scale and cognitive scores in dementia with Lewy bodies and Parkinson’s disease groups. Despite the reduction of substantia nigra [^18^F]-AV-1451 binding in dementia with Lewy bodies, these findings suggest that substantia nigra [^18^F]-AV-1451 binding has no value as a diagnostic marker in early Parkinson’s disease. Further investigations in longitudinal cohorts are warranted.

## Graphical abstract

**Graphical Abstract:**
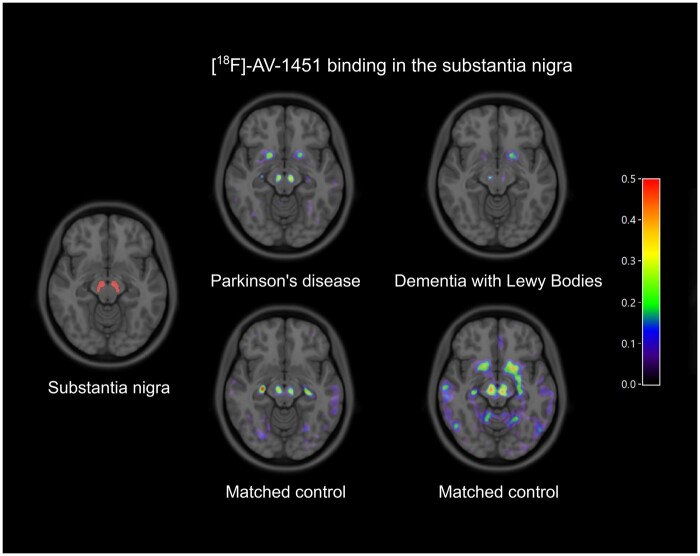

## Teaser

Mak and Kouli et al. evaluated [^18^F]-AV-1451 uptake of the substantia nigra in dementia with Lewy bodies and Parkinson’s disease patients. The findings do not strongly support [^18^F]-AV-1451 as a marker of pigmented neurons loss in the substantia nigra. Further longitudinal investigations in Lewy body diseases cohorts are warranted.

## Introduction

Parkinson’s disease and dementia with Lewy bodies (DLB) are neurodegenerative synucleinopathies that share the pathological hallmarks of Lewy bodies, neurites and alpha-synuclein inclusions. Both are characterized by early and progressive loss of dopaminergic neurons in the pars compacta of the substantia nigra. While Parkinson’s disease is primarily characterized by parkinsonism, DLB encompasses cognitive decline interfering with daily activities in association with (usually milder) parkinsonism.

There is a growing research effort to characterize the magnitude of dopaminergic neuron loss, and clarify the extent to which it is coupled with the clinical phenotype of DLB and Parkinson’s disease. To that end, PET and single photon emission computed tomography (SPECT) radiotracers have been developed to assess dopaminergic function at synaptic levels. However, no radiotracer has been established as a sensitive proxy of depigmentation in the substantia nigra. Post-mortem studies have demonstrated that the radioligand [^18^F]-AV-1451—originally developed to bind to hyperphosphorylated tau—shows off-target binding to neuromelanin-containing cells in the midbrain,^1^^,^^2^ leading to subsequent proposals that [^18^F]-AV-1451 binding in the substantia nigra may constitute an *in vivo* proxy of neuromelanin-containing cell density. Indeed, this was borne out by recent PET studies in Parkinson’s disease cases,^1–5^ with one study showing ∼30% decrease of substantia nigra [^18^F]-AV-1451 distribution volume [(uptake-1) × substantia nigra volume] in a modest sample of Parkinson’s disease subjects (*n* = 17) relative to controls (*n* = 16).^2^

However, the literature surrounding substantia nigra [^18^F]-AV-1451 binding has focussed on Parkinson’s disease, with inconsistent results (See Table 1 for a brief summary). One study showed a preservation of substantia nigra [^18^F]-AV-1451 binding in Parkinson’s disease without dementia, although Parkinson’s disease patients with dementia had decreased substantia nigra [^18^F]-AV-1451 binding.^5^ Furthermore, the clinical implications of substantia nigra [^18^F]-AV-1451 binding are still controversial, with some studies reporting associations with disease severity (MDS-UPDRS motor scale)^5^ and others not.^2^ Whether substantia nigra [^18^F]-AV-1451 uptake is reduced in DLB is also unclear, although Smith et al.^5^ did not find any significant differences, albeit in a small sample of 6 DLB patients. In addition to heterogeneity across the study samples (i.e. age, disease duration and the extent of cognitive and motor impairment), it is also possible that some of the discordant findings are attributable in part to methodological differences, such as the region of interest (ROI) definition and the metric used to quantify [^18^F]-AV-1451 in the substantia nigra.^2^^,^^3^

**Table 1:** Studies investigating substantia nigra [^18^F]-AV-1451 binding in PD and DLB cases

Author	Sample characteristics					Main finding
	Sample size	Age (Mean, SD)	Sex (M/F)	Duration (Mean, SD)	MDS-UPDRS-III (Mean, SD)	
Hansen et al., 2016^2^	17 PD	67.8 ± 6.5	13/4	5.2 (0.5–12.4)*	23.9 ± 12.6	30% reduction in [^18^F]-AV-1451 V_d_ in PD patients compared to controls. No correlation between nigral [^18^F]-AV-1451 V_d_ and disease duration or MDS-UPDRS-III motor score.
	16 Control	69.4 ± 7.3	12/4			
Cho et al., 2017^1^	15 PD	67.9 ± 5.4	8/7	5.5 ± 3.5	26.9 ± 11.2	13% lower [^18^F]-AV-1451 SUVR in PD patients compared to controls which did not survive region-wise multiple comparison. No correlation between [^18^F]-AV-1451 SUVR and disease duration or MDS-UPDRS-III motor score.
	15 Control	68.7 ± 5.3	8/7			
Coakeley et al., 2018^3^	6 PD	63.7 ± 9.6	3/3	5.5 ± 2.43	26.3 ± 3.01	Significant decrease in [^18^F]-AV-1451 SUVR in PD compared to healthy controls; mean SUVR was 21% lower in PD.
	10 Control	65.9 ± 9.9	2/8			
Schonhaut et al., 2017^4^	26 PD	67.1 ± 5.4	14/12	No information	26.1 ± 11.4	Lower [^18^F]-AV-1451 uptake (SUVR) in the substantia nigra in PD compared to controls.
	46 Control	69.6 ± 5.4	25/21			
Smith et al., 2018^5^	11 PD	67 ± 5.5	8/3	5.1 ± 4.0	11 ± 8	No difference between PD patients and controls, but a significant decrease in nigral [^18^F]-AV-1451 SUVR in PDD versus all other groups. This decrease correlated with higher MDS-UPDRS-III scores.
	18 PDD	73 ± 7.0	12/6	9.0 ± 4.1	27 ± 13	
	6 DLB	71 ± 6.3	3/3	5.5 ± 2.2	26 ± 21	
	44 Control	76 ± 5.3	21/23			

DLB, dementia with Lewy bodies; MDS-UPDRS-III, Movement Disorders Society Unified Parkinson’s disease rating scale part III (motor section); PD, Parkinson’s disease; PDD, Parkinson’s disease dementia; SUVR, Standardized uptake value ratio; *Mean (range); Vd, Distribution volume [(SUVR-1) × substantia nigra volume].

The objectives of this report are 2-fold. We test the hypothesis that nigral [^18^F]-AV-1451 binding is reduced in early-stage Parkinson’s disease, and in DLB, compared to controls. Unlike previous studies, we quantified [^18^F]-AV-1451 binding in the substantia nigra using non-displaceable binding potential (BP_ND_) determined from dynamic imaging rather than the standardized uptake value ratio (SUVR) or SUVR-based distribution volume^2^ from static imaging. We then test the hypothesis that motor and cognitive measures of disease severity correlate with [^18^F]-AV-1451 binding in the substantia nigra, as a biomarker of neuromelanin loss in early Parkinson’s disease and DLB.

## Materials and methods

### Participants and clinical data

There are four groups of participants in this study (Parkinson’s disease, DLB and two corresponding control groups) because the Parkinson’s disease and DLB projects were not performed on the same PET scanner. The Parkinson’s disease study was conducted on a PET-MR scanner and included 35 Parkinson’s disease cases from the NEuroinflammation and Tau accumulation in Parkinson’s Disease Dementia study (NET-PDD). The study was approved by the East of England—Essex Research Ethics Committee (16/EE/0445). Parkinson’s disease cases fulfilled UK PD Brain Bank Criteria for a diagnosis of Parkinson’s disease,^6^ were aged 55–80 with a disease duration of less than 2 years at the time of recruitment, and were recruited from the Parkinson’s Disease Research Clinic at the John Van Geest Centre for Brain Repair, University of Cambridge. Exclusion criteria were a diagnosis of dementia according to Movement Disorder Society PD-Dementia criteria^7^ and significant psychiatric disturbance. Thirty-eight age and sex-matched healthy controls were recruited from the NIHR Cambridge Bioresource (http://www.cambridgebioresource.org.uk) and the UK National Institute for Health Research Join Dementia Research register. One control was excluded due to a low score of 69 on the Addenbrooke's Cognitive Examination-III (ACE-III).

The second study was conducted on a PET-CT scanner, and included DLB cases and control subjects from the Neuroimaging of Inflammation in MemoRy and Other Disorders (NIMROD)^8^ and Tau Evaluation and Neurodegeneration in Dementia Research (TENDeR) studies. Participants over age 50 were recruited (10 DLB, 14 Controls). Patients were recruited from specialist memory clinics in and around Cambridgeshire, the Dementias and Neurodegeneration specialty of the UK Clinical Research Network (DeNDRoN) or the Join Dementia Research (JDR) platform (www.joindementiaresearch.nihr.ac.uk). Probable DLB was defined by both 2005 and 2017 consensus criteria.^9^^,^^10^ Healthy controls were recruited from the Join Dementia Research and local registers. The studies were approved by the East of England Ethics Committee (13/EE/0104; 16/EE/0529). The NET-PDD, NIMROD and TENDeR studies were all approved by the UK Administration of Radioactive Substances Advisory Committee.

### Clinical assessments

Participants underwent an initial assessment that included demographic measures, neuropsychological and cognitive testing (ACE-III for the Parkinson’s disease and corresponding control group or ACE-R for the DLB and corresponding control group), and evaluation of the severity of parkinsonism [MDS-Unified Parkinson’s Disease Rating Scale (MDS-UPDRS-III)]^11^ while the patients were ON medication.

### Structural MRI

Participants underwent an MRI session on a 3T scanner (Siemens Magnetom Tim Trio and Verio scanners; Siemens Healthineers, Erlangen, Germany) using a magnetization prepared rapid gradient echo (MPRAGE) T_1_-weighted sequence. The T_1_-weighted sequence (repetition time = 2300 ms, echo = 2.98 ms, field of view = 240 × 256 mm, 176 slices of 1 mm thickness, flip angle = 9°) was used to facilitate tissue class segmentation (grey and white matter, together with CSF), and to allow non-rigid registration of standard space ROIs to subject MRI space. Each T_1_-weighted image was non-rigidly registered to the ICBM 2009a template brain using ANTS (http://www.picsl.upenn.edu/ANTS/) and the inverse transform was applied to the standard space ROIs [a modified version of the n30r87 Hammersmith atlas (www.brain-development.org) resliced from MNI 152 to ICBM 2009a space] to bring the ROIs to subject MRI space.

### PET imaging of [^18^F]-AV-1451

All participants underwent [^18^F]-AV-1451 PET to assess the extent and magnitude of brain tau pathology (PET-MR: Parkinson’s disease and controls; PET-CT: DLB and controls). [^18^F]-AV-1451 was prepared at the Wolfson Brain Imaging Centre, University of Cambridge, with high radiochemical purity (>95%) and a specific activity of 216 ± 60 GBq/μmol at the end of synthesis. PET scanning for the DLB cases and their controls was performed on a GE Advance PET scanner or a GE Discovery 690 PET/CT (GE Healthcare, Waukesha, USA). A 15-min ^68^Ge/^68^Ga rotating rod transmission scan was used for attenuation correction on the Advance, which was replaced by a low dose CT scan on the Discovery 690. The emission protocols were the same on both scanners: 90 min dynamic imaging following a 370 MBq [^18^F]-AV-1451 injection. The Parkinson’s disease cases and matched controls underwent 90 min dynamic [^18^F]-AV-1451 PET imaging on a GE SIGNA PET/MR scanner. Owing to the higher sensitivity of the SIGNA PET/MR, the target injection activity was reduced to 185 MBq. Attenuation correction for the PET/MR data included the use of a multi-subject atlas method^12^^,^^13^ and improvements to the MRI brain coil component.^14^ The dynamic PET data were histogrammed into the same time frames for both scanners. Image reconstruction on the PET-CT scanner used the PROMIS 3D filtered back projection algorithm,^15^ whereas time-of-flight ordered subsets expectation maximization^16^ with 6 iterations and 16 subsets was used on the PET/MR. In all cases, corrections were applied for randoms, dead time, normalization, scatter, attenuation and sensitivity. Each emission image series was aligned using SPM12 (www.fil.ion.ucl.ac.uk/spm/software/spm12) to ameliorate the effect of patient motion during data acquisition. The mean aligned PET image, and hence the corresponding aligned dynamic PET image series, was rigidly registered to the T_1_-weighted image using SPM12 to extract radioactivity concentration values from both the Hammersmith atlas ROIs and the reference region in cerebellar grey matter. All ROI time-activity curves, including that for the reference tissue, were corrected for CSF partial volume error through division with the ROI grey matter plus white matter tissue fraction obtained from SPM12 probability maps smoothed to PET spatial resolution. [^18^F]-AV-1451 non-displaceable binding potential (BP_ND_), a measure of specific binding, was determined for the substantia nigra with a basis function implementation of the simplified reference tissue model.^17^

### Statistical analyses

Statistical analyses were performed in R. Group differences in continuous variables were assessed with two-tailed Student’s *t*-test or Wilcoxon rank-sum test depending on the normality of the data. The chi-square test was used to compare categorical data between the groups. ANCOVA, adjusting for age, was used to perform group comparisons of substantia nigra [^18^F]-AV-1451 BP_ND_. Linear regressions were used to examine correlations of the substantia nigra [^18^F]-AV-1451 BP_ND_ with age, disease duration, MDS-UPDRS-III, and ACE-R/ACE-III scores in the DLB and Parkinson’s disease groups. *P*-values < 0.05 were considered statistically significant.

### Data availability

The derived data that support the findings of this study are available from the corresponding author, upon reasonable request for academic (non-commercial) purposes.

## Results

### Sample characteristics

Demographics and clinical variables are summarized in Tables 2 and 3. Parkinson’s disease cases and their controls did not differ in terms of age (*P* = 0.47), sex distribution (*P* = 0.22) and education years (*P* = 0.24). Relative to controls, DLB subjects were significantly older (*P* < 0.01), had less years of education (*P* < 0.01), but did not differ in terms of sex distribution (*P* = 0.22). Both the Parkinson’s disease and DLB groups had lower cognitive scores compared to controls (*P* < 0.05).

**Table 2:** Demographics and clinical variables of PD cases and corresponding controls

	HC (*N* = 37)	PD (*N* = 35)	*P*-value
Age (Years)			
Mean (SD)	67.5 (7.87)	66.5 (6.95)	0.572
Median [Min, Max]	70.6 [51.0, 84.0]	67.1 [52.5, 80.0]	
Sex			
Female	17 (45.9%)	11 (31.4%)	0.307
Male	20 (54.1%)	24 (68.6%)	
Education (Years)			
Mean (SD)	15.0 (3.65)	13.7 (2.78)	0.11
Median [Min, Max]	16.0 [10.0, 25.0]	13.0 [10.0, 18.0]	
ACE-III			
Mean (SD)	96.6 (2.83)	93.0 (4.58)	<0.001
Median [Min, Max]	97.0 [88.0, 100]	95.0 [82.0, 100]	
MDS-UPDRS-III			
Mean (SD)		28.5 (10.0)	
Median [Min, Max]		28.5 [10.0, 46.0]	
Disease duration (Years)			
Mean (SD)		1.08 (0.581)	
Median [Min, Max]		0.900 [0.362, 2.34]	

ACE-III, Addenbrooke's Cognitive Examination-III; HC, healthy controls; MDS-UPDRS-III, Movement Disorders Society-Unified Parkinson's Disease Rating Scale part III; PD, Parkinson’s disease.

**Table 3:** Participant characteristics of DLB cases and corresponding controls

	HC (*N* = 14)	DLB (*N* = 10)	*P*-value
Age (Years)			
Mean (SD)	67.4 (8.21)	78.3 (6.00)	0.001
Median [Min, Max]	69.0 [55.0, 80.0]	77.0 [71.0, 86.0]	
Sex			
Female	7 (50.0%)	2 (20.0%)	0.285
Male	7 (50.0%)	8 (80.0%)	
Education (Years)			
Mean (SD)	15.8 (1.93)	11.7 (2.58)	<0.001
Median [Min, Max]	16.0 [11.0, 19.0]	10.5 [9.00, 17.0]	
ACE-R			
Mean (SD)	93.6 (4.69)	73.6 (19.6)	0.011
Median [Min, Max]	95.0 [82.0, 99.0]	76.0 [36.0, 96.0]	
MDS-UPDRS-III			
Mean (SD)		25.0 (11.7)	
Median [Min, Max]		26.0 [11.0, 52.0]	
Disease duration (Years)			
Mean (SD)		2.10 (1.44)	
Median [Min, Max]		2.14 [0.0904, 3.90]	

ACE-R, Addenbrooke's Cognitive Examination-Revised; DLB, dementia with Lewy bodies; HC, healthy controls; MDS-UPDRS-III, Movement Disorders Society-Unified Parkinson's Disease Rating Scale part III.

### Comparisons of substantia nigra [^18^F]-AV-1451 binding

**Figure 1:**
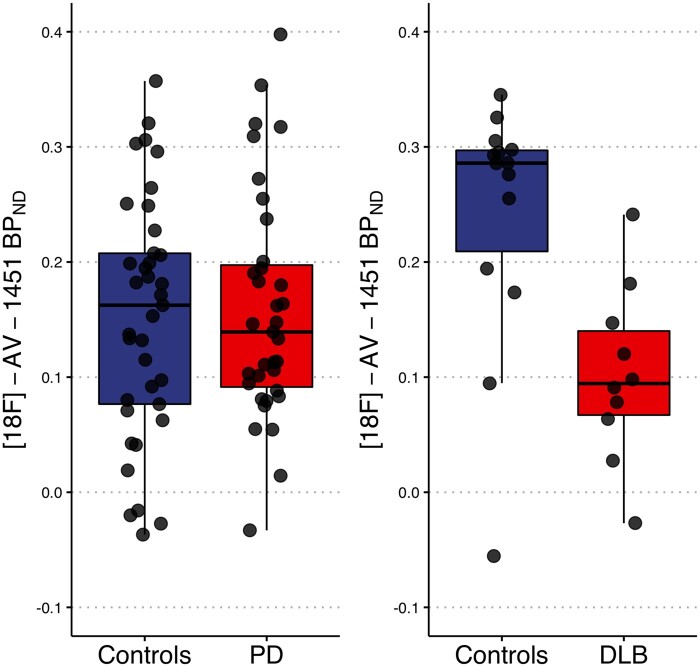
[^18^F]-AV-1451 binding potential (BP_ND_) in the substantia nigra is reduced in DLB (ANCOVA: *F* = 5.4, *P* = 0.03) but not in PD (ANCOVA: *F* = 0.06, *P* = 0.81). DLB, Dementia with Lewy bodies; PD, Parkinson’s disease.

Substantia nigra [^18^F]-AV-1451 BP_ND_ was not significantly different between Parkinson’s disease and controls (Fig. 1). In contrast, the DLB group showed significantly reduced substantia nigra [^18^F]-AV-1451 BP_ND_ compared to controls (age adjusted *P* = 0.03; Fig. 1).

### Associations of substantia nigra [^18^F]-AV-1451 binding with clinical variables

**Figure 2:**
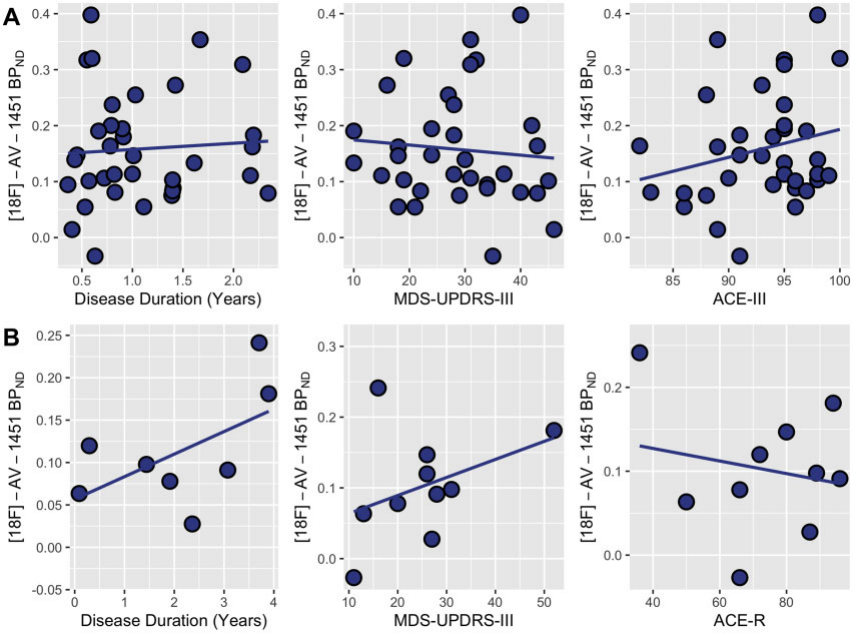
**Scatterplots of the relationship between [^18^F]-AV-1451 binding in the substantia nigra and disease duration, motor severity (MDS-UPDRS-III) or cognitive severity (ACE-III/ACE-R) in** (**A**) PD and (**B**) DLB. ACE-III, Addenbrooke’s Cognitive Examination-III; ACE-R, Addenbrooke's Cognitive Examination-Revised; DLB, Dementia with Lewy bodies; MDS-UPDRS-III, Movement Disorders Society-Unified Parkinson's Disease Rating Scale part III; PD, Parkinson’s disease.

Linear regressions with substantia nigra [^18^F]-AV-1451 BP_ND_ as the predictor variable revealed no significant associations with any clinical variables in either DLB or Parkinson’s disease groups (Fig. 2).

## Discussion

The availability of a PET radiotracer to quantify the number of neuromelanin-containing cell in the substantia nigra could facilitate tracking of disease progression and treatment outcomes in future clinical trials for people with Parkinson’s disease and DLB. In our study, DLB subjects showed significantly reduced substantia nigra [^18^F]-AV-1451 binding compared to controls. However, substantia nigra [^18^F]-AV-1451 binding was comparable between early Parkinson’s disease cases and controls, and not associated with disease duration, cognitive and motor function in either Parkinson’s disease or DLB groups.

Our observation of reduced substantia nigra [^18^F]-AV-1451 binding in DLB has extended results from recent studies in Parkinson’s disease.^2^^,^^5^^,^^18^ The magnitude of substantia nigra [^18^F]-AV-1451 binding reduction in our DLB patients was ∼55% in comparison to ∼30% reduction among Parkinson’s disease patients without dementia.^2^ Interestingly, it has been shown that a 45% loss of dopaminergic neurons is commensurate with Braak alpha-synuclein stage III.^19^ While our finding is in keeping with previous reports, it would be critical to assess the extent to which substantia nigra [^18^F]-AV-1451 binding correlates with longitudinal clinical decline in DLB. Correlations of substantia nigra [^18^F]-AV-1451 binding with striatal dopamine transporter signal on [^123^I]-FP-CIT scans would also be informative.

In contrast, we did not replicate the significant substantia nigra [^18^F]-AV-1451 uptake reductions previously reported in Parkinson’s disease.^1–4^ These inconsistencies may relate to disease duration; the average disease duration in our Parkinson’s disease cohort was just 1.5 years, considerably shorter than that of Coakeley et al. (5.5 years) and Hansen et al. (5.2 years). Given the reported correlation between substantia nigra [^18^F]-AV-1451 distribution volume [i.e. (SUVR-1) × substantia nigra volume] and disease duration,^5^ it is conceivable that reduced substantia nigra [^18^F]-AV-1451 binding is only detectable in later-stage Parkinson’s disease, with the loss of striatal dopaminergic terminal function occurring before the loss of neuronal cell bodies in the substantia nigra. However, a reduction in binding even at the time of diagnosis might be expected given that approximately 30–50% of dopaminergic neurons in the substantia nigra are estimated to be lost when Parkinson’s disease motor symptoms begin. Despite showing a significant reduction of substantia nigra [^18^F]-AV-1451 distribution volume in Parkinson’s disease, Hansen et al.^2^ acknowledged there was a considerable overlap between the Parkinson’s disease and controls. Taken together, these findings suggest that substantia nigra [^18^F]-AV-1451 binding has no value as a diagnostic marker in early Parkinson’s disease.

The extent to which substantia nigra [^18^F]-AV-1451 binding is related to clinical measures is another topic of uncertainty, with conflicting evidence from small samples.^2^^,^^5^ In the largest sample of DLB and Parkinson’s disease subjects to date, our data do not indicate a relationship between substantia nigra [^18^F]-AV-1451 binding and disease duration, cognition or MDS-UPDRS motor scores. Therefore, our findings do not lend strong support for the use of substantia nigra [^18^F]-AV-1451 PET to monitor disease duration and/or severity, and by extension, for its utility as a biomarker for assessing efficacy in future neuro-protective drug trials.

Previous studies of [^18^F]-AV-1451 binding in the substantia nigra have used SUVR as a metric of binding. BP_ND_ is a more accurate measure of specific binding than SUVR-1 as it is much less affected by the confounding effect of variable tracer delivery. The accuracy with which SUVR-1 estimates BP_ND_ is also affected by the level of tracer binding in the tissue as this affects the optimal time window for SUVR estimation. Given the above, we used BP_ND_ estimated from dynamic imaging rather than SUVR from static imaging as a metric of [^18^F]-AV-1451 binding in the substantia nigra.

Our findings should be interpreted with several caveats. The lack of a reduction in substantia nigra [^18^F]-AV-1451 binding is limited to early-stage Parkinson's disease and we could not rule out an effect at later disease stages or in a group with a larger range of disease duration. The sample size of our DLB group was limited; our DLB and control group were not age-matched (although age was adjusted for in our analyses, and we showed a lack of correlation between age and substantia nigra [^18^F]-AV-1451 binding; Supplementary Table 1); and the study design was cross-sectional. The finding of reduced substantia nigra [^18^F]-AV-1451 binding in DLB should be validated in future longitudinal studies. In addition, substantia nigra [^18^F]-AV-1451 binding could be cross-validated through direct comparison to other *in vivo* proxies of neuromelanin. One promising candidate for such cross-validation work is novel MRI sequences of neuromelanin,^20^ which have found decreases in the neuromelanin MRI signal in Parkinson’s disease relative to controls ^21^ as well as associations with the activity of midbrain dopamine neurons.^22^ Finally, in the context of its potential role as an outcome marker in clinical trials, studies are also needed to assess the test–retest reproducibility of substantia nigra [^18^F]-AV-1451 binding, and establish the longitudinal rate of loss of substantia nigra [^18^F]-AV-1451 binding in normal ageing as well as DLB.

The availability of a radiotracer to serve as a measure of the pigmented dopaminergic neuronal count of the substantia nigra would facilitate our efforts to understand the relationship between dopaminergic deficits and clinical decline in Lewy body diseases. Our findings do not support the interpretation of [^18^F]-AV-1451 as a generic marker of the loss of pigmented neurons in the substantia nigra of patients with Lewy body disorders, but further longitudinal investigations in Lewy body diseases cohorts are warranted.

## Supplementary material

Supplementary material is available at *Brain Communications* online.

## Supplementary Material

fcab177_Supplementary_Data

## Abbreviations

ACE-III =: Addenbrooke’s Cognitive Examination-III
ACE-R =: Addenbrooke’s Cognitive Examination-Revised
BP_ND_ =: non-displaceable binding potential
DLB =: dementia with Lewy bodies
MDS-UPDRS =: Movement Disorders Society—Unified Parkinson’s Disease Rating Scale
ROI =: region of interest
SPECT =: single photon emission computed tomography
SUVR =: standardized uptake value ratio
