# Supplementary material for: [18F]-AV-1451 binding in the substantia nigra as a marker of neuromelanin in Lewy body diseases
Source: Brain Commun. 2021 Aug 28;3(3):fcab177. doi: 10.1093/braincomms/fcab177 (PMC8410984; doi:10.1093/braincomms/fcab177)
Supplement: fcab177_Supplementary_Data [file fcab177_supplementary_data.docx]

| **Groups** | **T-Statistic** | **P value** |
| --- | --- | --- |
| PD | -0.10 | 0.92 |
| DLB | -0.59 | 0.57 |
| PD controls | -0.55 | 0.59 |
| DLB controls | -0.64 | 0.53 |

**Supplementary Table 1**. Associations of substantia nigra [^18^F]-AV-1451 BP_ND_ with age using linear regression.
